# Inhibition of Human Neutrophil Elastase by Pentacyclic Triterpenes

**DOI:** 10.1371/journal.pone.0082794

**Published:** 2013-12-20

**Authors:** Li Feng, Xiaoyu Liu, Weiliang Zhu, Fujiang Guo, YingchunWu, Rui Wang, Kaixian Chen, Cheng Huang, Yiming Li

**Affiliations:** 1 School of Pharmacy, Shanghai University of Traditional Chinese Medicine, Shanghai, China; 2 Department of Biological Chemistry, Second Military Medicinal University, Shanghai, China; 3 Shanghai Institute of Materia Medica, Chinese Academy of Sciences, Shanghai, China; University of Rochester Medical Center, United States of America

## Abstract

**Scope:**

Inhibiting human neutrophil elastase (HNE) is a promising strategy for treating inflammatory lung diseases, such as H1N1 and SARS virus infections. The use of sivelestat, the only clinically registered synthesized HNE inhibitor, is largely limited by its risk of organ toxicity because it irreversibly inhibits HNE. Therefore, potent reversible HNE inhibitors are promising alternatives to sivelestat.

**Methods and Results:**

An *in vitro* HNE inhibition assay was employed to screen a series of triterpenes. Six pentacyclic triterpenes, but not tetracyclic triterpenes, significantly inhibited HNE. Of these pentacyclic triterpenes, ursolic acid exhibited the highest inhibitory potency (IC_50_ = 5.51 µM). The HNE inhibitory activity of ursolic acid was further verified using a mouse model of acute smoke-induced lung inflammation. The results of nuclear magnetic resonance and HNE inhibition kinetic analysis showed that the pentacyclic triterpenes competitively and reversibly inhibited HNE. Molecular docking experiments indicated that the molecular scaffold, 28-COOH, and a double bond at an appropriate location in the pentacyclic triterpenes are important for their inhibitory activity.

**Conclusion:**

Our results provide insights into the effects of pentacyclic triterpenes on lung inflammatory actions through reversible inhibition of HNE activity.

## Introduction

Neutrophils are major mediators of inflammation. They are involved in the pathogenesis of various lung inflammatory diseases, including acute lung injury (ALI), acute respiratory distress syndrome (ARDS), bronchiectasis, pulmonary emphysema, and chronic obstructive pulmonary disease (COPD) [1]. For example, the number of airway neutrophils is an important indicator for ALI and ARDS [2]. Furthermore, the percentage of airway neutrophils in bronchial tissues is related to the severity of airflow obstruction in COPD [3]. Neutrophilic inflammation attracted considerable attention because of influenza caused by H1N1 or SARS virus [4].

Human neutrophil elastase (HNE, E.C. 3.4.21.37) is a 30 kDa serine protease stored in the azurophilic granules of neutrophils. Intracellular HNE breaks down foreign proteins (e.g., those from invading bacteria), whereas the extracellular HNE released by neutrophils and mostly bound to the neutrophil plasma membrane, assists neutrophil migration to inflammation sites by degrading various host proteins, such as extracellular matrix proteins [5]. Active HNE is detectable at inflammation sites even though endogenous inhibitors are present in the plasma. For example, active HNE is detectable in the bronchoalveolar lavage fluid (BALF) of COPD patients and its activity corresponds to the level of inflammation [6]. Various studies have shown that HNE regulates local inflammatory processes. In turn, inflammatory cytokines, such as tumor necrosis factor-α (TNF-α), interleukin-1β (IL-1β), IL-2, IL-6, and IL-8, activate neutrophils, causing excessive HNE release [7], [8].

Under normal physiologic conditions, HNE is controlled by its endogenous inhibitors, including α_1_-antitrypsin (α_1_-AT), secretory leukocyte proteinase inhibitor, α_2_- macroglobulin, and elafin [9]–[12]. However, large amounts of oxygen radicals and proteases released by leukocytes recruited to inflammation sites can inactivate these endogenous inhibitors [13]. Moreover, the tight binding of extracellular HNE to the neutrophil membrane can restrict circulating endogenous inhibitors [14]. Thus, an imbalance between HNE and its endogenous inhibitors can stimulate inflammatory lung disorders because of the involvement of HNE in inflammation, mucus overproduction, and lung tissue damage [15]–[17].

Sivelestat sodium hydrate (ONO-5046) is the only clinically registered chemically synthesized selective HNE inhibitor. It attenuates pulmonary disorders and improves pulmonary function. ONO-5046 is clinically used to treat pneumonia and ALI caused by viral infections [4]. However, the use of ONO-5046 is limited by its poor pharmacokinetics and potential risks of organ toxicity because it irreversibly inhibits HNE by covalently binding to Ser-195 [1], [18], [19].

Natural compounds are a potential source of HNE inhibitors. Some natural compounds reportedly inhibit HNE activity *in vitro* [5]. However, the structure–activity relationship, inhibitory mechanism, and physiologic effects of these molecules *in vivo* remain unclear.

In the present study, we used molecular docking to analyze the binding capabilities of HNE with a series of compounds isolated from herbs that inhibit lung inflammation. We subsequently confirmed their inhibitory activity *in vitro* and *in vivo*. The results indicated that the compounds with a pentacyclic triterpene scaffold significantly inhibit HNE through reversible competitive binding mode, whereas tetracyclic triterpenes did not exhibit notable inhibitory activity. The molecular scaffold, the carboxyl function at position 28, and a double bond at the appropriate location in the pentacyclic triterpenes are crucial to their inhibitory activity.

Pentacyclic triterpenes, such as ursolic acid and oleanolic acid, are widely distributed in apples and other fruits; these compounds are used in nutraceuticals for treating various diseases [20], [21]. Our results provide insights into the potential of pentacyclic triterpenes as dietary supplements and functional food ingredients.

## Materials and Methods

### Materials and Test Compounds

HNE (EC 3.4.21.37) from human leukocytes was purchased from Innovative Research Company (Novi, Michigan). ONO-5046, HNE substrate (MeO-Suc-Ala-Ala -Pro-Val-pNA), soybean trypsin inhibitor, and DMSO were obtained from Sigma (St. Louis, MO, USA) and used without further purification. The Mouse NE ELISA Kit (96T, Catalog Number: CSB-E04804m) and Mouse TNF-α ELISA MAX™ Deluxe Set (Lot Number: B138014) were purchased from Cusabio (Wuhan, China) and Biolegend (California, USA), respectively. All other reagents were of analytical grade.

**Figure 1:**
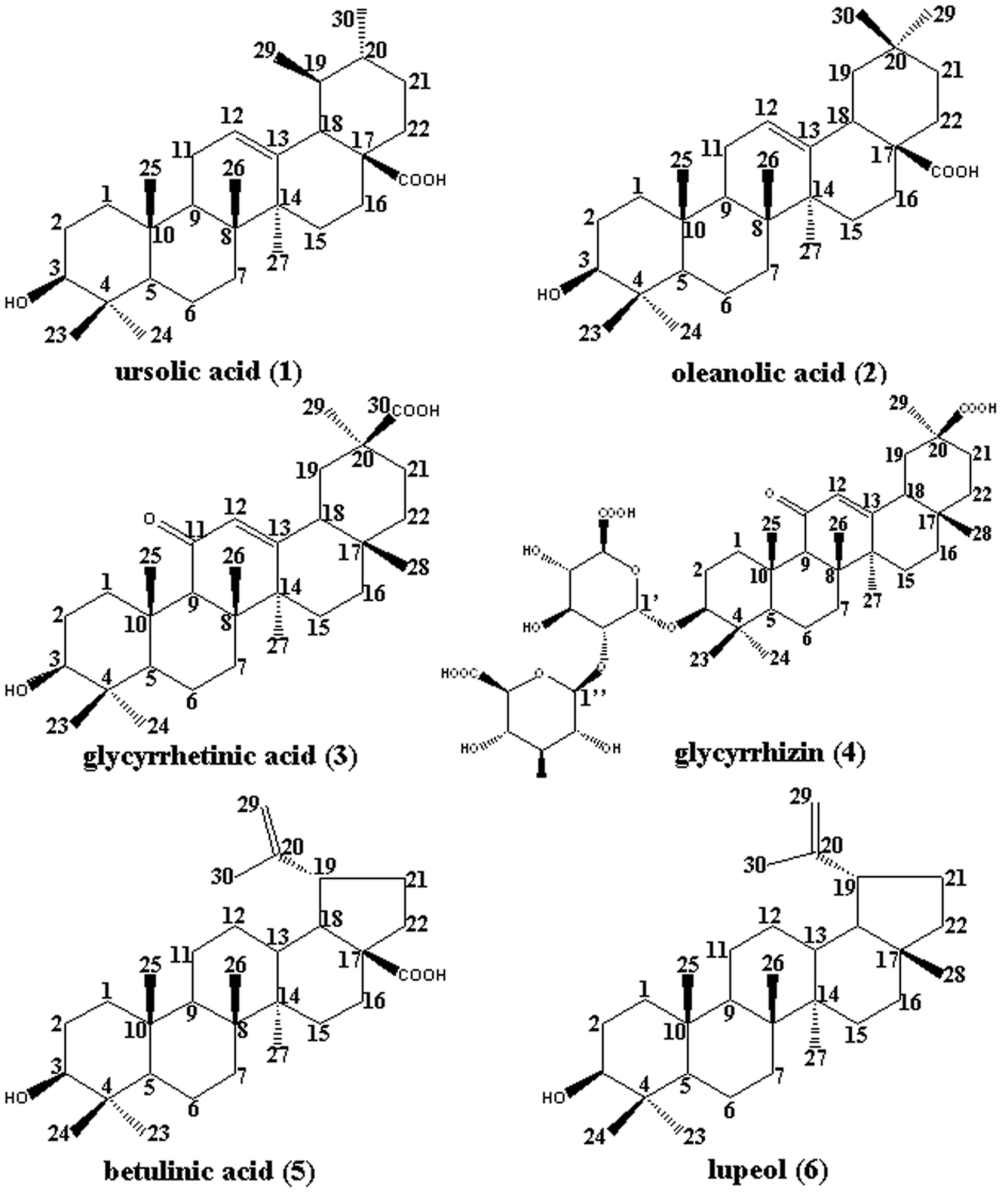
Chemical structures of pentacyclic triterpenes 1 to 6.

**Figure 2:**
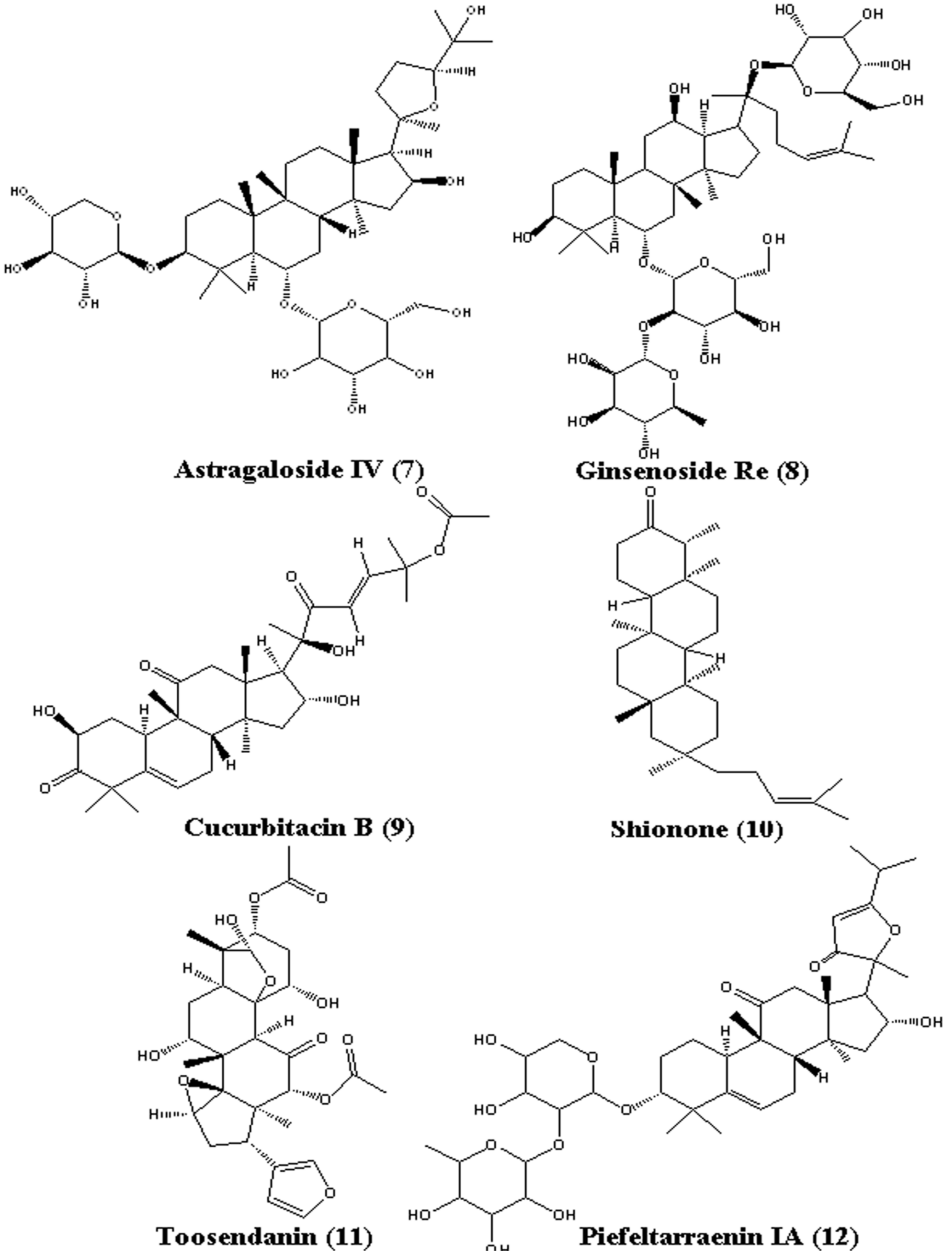
Chemical structures of tetracyclic triterpenes 7 to 12.

**Figure 3:**
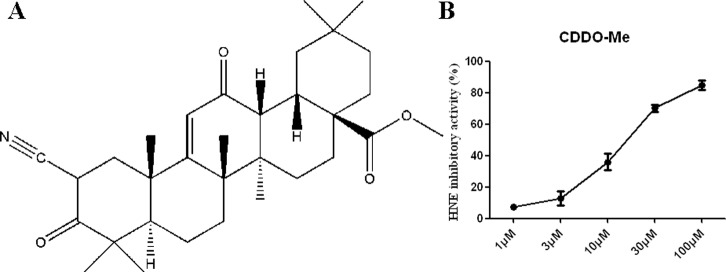
Chemical structure (A) and anti-HNE activity (B) of CDDO-Me.

Pentacyclic triterpene compounds **1** to **6** (**Fig. 1**), tetracyclic triterpene compounds **7** to **12** (**Fig. 2**), and other tested natural compounds were obtained from the Shanghai R&D Center for Standardization of Chinese Medicines (Shanghai, China). The C-28 methyl ester of 2-cyano-3,12-dioxoolen-1,9-dien-28-oic acid (CDDO-Me, **Fig. 3**) was bought from Shanghai Boyle Chemical Co., Ltd. (Shanghai, China). The compounds were authenticated through their^ 1^H-NMR spectra. Their purity was determined to exceed 98% through HPLC/MS.

### Molecular Docking

The crystal structure (PDB ID: 1B0F) of HNE in complex with the inhibitor SEI was downloaded from the RCSB protein data bank [22]. According to the Amber force field, the potential 3D structure of HNE was assigned with Kollman united atom charges encoded using the molecular modeling software Sybyl 7.3 (Linux-os2x). Sybyl 7.3 was also used to distribute the essential hydrogen atoms of HNE. The structures of all test compounds were obtained from the PubChem Compound database (http://www.ncbi.nlm.nih.gov/pccompound). The geometries of all compounds were optimized by applying Gasteiger–Marsili atom charges [22].

The advanced docking program Autodock 3.0.3 was used to perform automated molecular docking to handle the interaction mode of the triterpenes with HNE [23], [24]. The Lamarckian genetic algorithm was used to describe the relationship between the inhibitors and the enzymes through their orientation, translation, and the conformation of their inhibitors [25]. The number of energy evaluations was set to 250000, their population size was set to 50, their generations was set to 27000, and their docking runs was set to 30, based on the requirements of the Amber force field. The root mean square deviation between the conformational superposition of SEI from 1B0F crystal structure and that from the docking result was 0.44 Å, which suggests that the parameter set for the AutoDock simulation was reasonable for reproducing the X-ray structure. The triterpene structures of the training set were manually docked into the binding pocket of the HNE enzyme. Each docking cycle consisted of a fitness evaluation, cross-over, mutation, and selection. A Solis and Wets local search was used to implement the energy minimization on a user-specified proportion of the population [26]. The optimal conformations of the HNE–ligand complex were handled using Sybyl 7.3, and their 3D figures were viewed using PyMol software.

### HNE Activity Inhibitory Bioassay

The direct inhibition assay of HNE activity was performed as previously described [27], with ONO-5046 as the positive control [22]. Briefly, 200 µL of the substrate solution (1.4 mM MeO-Suc-Ala-Ala-Pro-Val-pNA in 10 mM Tris-HCl buffer, pH 7.5) was mixed with different concentrations of the test compound solution. The triterpenes stock solutions were dissolved in DMSO, which was restricted to 0.25% and diluted with Tris-HCl buffer to produce the final sample concentrations. Up to 1 µL of enzyme solution (0.01 U of HNE) was added to the solution, and the resultant mixture was incubated for 1 h at 37°C in the dark. Subsequently, the reaction was quenched by adding 200 µL of soybean trypsin inhibitor at a final concentration of 0.2 mg/mL. The rate of enzymatic hydrolysis of the substrate was followed by increased absorbance at 405 nm because of the release of 4-nitroaniline. The color intensity of the digested substrate was then immediately measured at 405 nm using an automatic microplate reader (Bio-Tek, Synergy HT).

The enzyme inhibition kinetic analysis was performed by maintaining the ursolic acid concentration (0, 3, and 10 µM) and using six different HNE substrate concentrations (0.2 mM to 2 mM) in a manner analogous to the assay described above. The UV absorption at 405 nm was monitored for 5 min using an automatic microplate reader. The apparent maximum velocity (V_max_′) and the apparent Michaelis–Menten constant (K_app_) were determined by fitting the initial velocity at each HNE substrate concentration [28].

The absorbance data are expressed as mean ± standard deviation. The calculations were carried out in triplicate using three separate experiments. The data were statistically analyzed via one-way ANOVA, followed by a Dunnett’s *t*-test using SPSS 16.0. Differences in K_app_ and V_max_′ were tested using GraphPad Prism 5 software. Differences with P<0.05 were considered statistically significant.

### Activity of Triterpenes in Acute Smoke-induced Lung Inflammation Model in Mice

#### Animals

The animal study protocols were validated and approved by the Shanghai University of Traditional Chinese Medicine (2013008) and were carried out in accordance with internationally accepted guidelines. Female BALB/c mice (18 g to 20 g) were obtained from the SLAC Laboratory (Shanghai, China). All mice were kept under a 12-hour light–dark cycle, temperature of 22°C to 23°C, and humidity of 50% to 60%.

The mouse model of acute smoke-induced airway inflammation was established to assay the activity of triterpenes *in vivo* [29]–[31]. The mice were allowed 1 week to adapt to the standard housing conditions. After health monitoring, the mice were placed in individual wire cages, which were placed inside a closed plastic box (volume: 24 cm×26 cm×40 cm) connected to the smoke source. Mainstream cigarette smoke was obtained by smoking 12 consecutive Honghe cigarettes (12 mg tar, 1.1 mg nicotine, and 13 mg CO per cigarette) according to the Federal Trade Commission protocol (1 puff/min of 2 seconds duration and 35 mL volume) via a peristaltic pump (HL-1, Qingpu Huxi, Shanghai) with some modifications [32]. The exposure was performed for 10 min on day 1, 30 min on day 2, and 50 min on day 3 to enable the mice to acclimatize to the new smoking conditions. From day 4, the mice were placed in the smoke exposure box and subjected two 50 min exposures with 50 min intervals (taken out to fresh air) every day for five consecutive days.

Ursolic acid (**1**), astragaloside IV (**7**), and ONO-5046, which was used as the positive control in the vehicle (20% DMSO in double distilled water), were intraperitoneally injected into the mice 45 min before smoke exposure. The control mice, which were exposed to fresh air and smoke without drug treatment, were administered the vehicle 45 min before exposure to fresh air or smoke. The mice were sacrificed 16 h after the final smoke exposure through an overdose of urethane, and their BALF was analyzed. Total and differential BALF cell counts were performed, and the NE and TNF-α in the BALF samples were analyzed using a Mouse NE ELISA Kit (Cusabio, Wuhan, China) and Mouse TNF-α ELISA MAX™ Deluxe Set (Biolegend, California, USA), respectively.

In this acute smoke model, data are presented as mean ± standard error, and the statistical differences among air, smoke vehicle, and smoke with different compound treatment groups were analyzed using a one-way ANOVA followed by an LSD test for multiple comparisons.

### NMR Spectroscopy

Stock solutions of pentacyclic triterpenes and HNE were dissolved in d_6_-DMSO and 50 mM phosphate buffer, respectively, after which the latter was gradually added to the former. Solution pH was carefully adjusted to 7.0 using either DCl or NaOD. Finally, the sample for the NMR study was prepared in 99.9% D_2_O buffered at pH 7.0 (10 mM phosphate buffer) containing 80% d_6_-DMSO. The concentrations of the tested compounds and HNE were 1.0 mM and 5.0 µM, respectively. All measurements were performed on a Bruker Advance 400 MHz NMR spectrometer at 298 K. The suppression of residual water signals was achieved through the Watergate pulse program with gradients.

^1^H NMR spectra were recorded using a BBO broadband probe. A total of 64 scans were collected into 4 K data points at a spectral width of 4000 Hz. The spin-lattice relaxation rates were measured using the (180°-τ−90°-t)_n_ sequence. The τ values used for the selective and nonselective experiments were as follows: 0.00001, 0.001, 0.01, 0.1, 0.2, 0.3, 0.4, 0.5, 0.6, 0.7, 0.8, 1, 1.2, 1.5, 2, 3, and 5.0 s, and the delay time (t) was 0.5 s. The 180° selective inversion of the proton spin population was obtained via a 20 ms selective Gaus1_180i.1000 shape pulse at 50 dB; these measurements correspond to an excitation width of approximately 45 Hz [33]. All relaxation rates were calculated under initial rate approximation [34]. All NMR data were processed using Bruker Topspin 2.1 software.

## Results

### Binding Features of Triterpenes with HNE, as Detected using Molecular Docking

A total of 323 compounds from 11 herbs (**Table S1** **in File S1**) that protect lung function according to traditional Chinese medicine [35], [36] were docked with HNE to form ligand–enzyme complexes using Autodock 3.0.3 software. The docked complexes were selected based on the criteria on geometrical matching quality combined with interaction energy. Subsequently, 30 docked conformations of each ligand were analyzed in terms of hydrogen bonding, hydrophobic interaction, and the free binding energy between the ligand and the protein. The conformation that corresponds to the lower binding energy and the key amino acid residues in the HNE interaction with the ligand was selected as the best binding conformation. The optimal conformation of the HNE–ligand complex was handled using Sybyl 7.3. The hydrophobic interactions and hydrogen bonding interactions between the ligands and HNE were observed using PyMol software. A total of 57 compounds with potential HNE inhibitory activity are shown in **Table S2** (in **File S1**).

### Triterpene Inhibition of HNE *in vitro*

The inhibitory effects of the compounds selected through molecular docking and our other available compounds were further investigated using an HNE *in vitro* activity assay. ONO-5046, the irreversible HNE inhibitor, with an IC_50_ value of 87.05 nM, was used as the positive control [22]. HNE activity was strongly inhibited by the six natural pentacyclic triterpenes (compounds **1** to **6**) in a dose-dependent manner (**Table 1**). In this assay, ursolic acid, oleanolic acid, betulinic acid and lupeol of the six pentacyclic triterpenes inhibited HNE activity by more than 80% at 30 µM (p<0.01). Ursolic acid (**1**) showed the most potent inhibitory activity among the six pentacyclic triterpenes, with a minimal inhibition (12.2%) at 1 µM and a maximal inhibition (>80%) at >10 µM. Consistently, the IC_50_ values of the six natural pentacyclic triterpenes were approximately 5.51 µM (ursolic acid), 8.05 µM (oleanolic acid), 36.90 µM (glycyrrhetinic acid), 27.58 µM (glycyrrhizin), 6.83 µM (betulinic acid), and 8.20 µM (lupeol). Considering ursolic acid exhibited the highest inhibitory activity against HNE, it was further studied in a subsequent *in vivo* bioassay.

**Table 1:** Anti-HNE activity of six selected pentacyclic triterpenes (mean ± SD), n = 3.

Compound/C	1 µM	3 µM	10 µM	30 µM	100 µM
**1**	12.22±6.64	36.47±5.75**	80.84±3.14**	82.98±5.67**	88.47±2.96**
**2**	–2.28±5.71	1.66±3.61	53.44±8.62**	83.98±3.59**	88.14±3.72**
**3**	0.06±6.40	1.05±5.24	21.09±4.96	45.84±0.41**	75.20±2.89**
**4**	10.25±1.73	19.00±6.99*	28.21±4.84**	40.89±1.28**	78.66±1.99**
**5**	8.25±1.88	46.57±2.47**	74.20±2.32**	80.58±2.21**	82.41±1.37**
**6**	9.38±6.80	19.01±10.00*	51.92±15.22**	88.00±6.26**	93.56±1.19**

Inhibitory ratios are expressed as mean ± standard error.

* P<0.05 vs. the control group.

** P<0.01 vs. the control group.

The inhibition of HNE by the pentacyclic triterpenes identified from natural products prompted us to evaluate CDDO-Me. CDDO-Me, a synthetic pentacyclic triterpene based on naturally occurring ursolic and oleanolic acids, inhibits inflammation and the development of emphysema in mice chronically exposed to cigarette smoke [37]–[39]. Our current results showed that CDDO-Me significantly inhibited HNE activity, with an IC_50_ of 16.59 µM (**Fig. 3**), which suggest that the anti-inflammation effects of CDDO-Me are mediated, at least partly, through the inhibition of HNE activity.

In contrast to the inhibitory potencies of pentacyclic triterpenes, the six tested tetracyclic triterpenes (compounds **7** to **12**) minimally affected HNE activity at concentrations up to 100 µM (**Table S3 in File S1**). This result indicates that the pentacyclic triterpene scaffold is important for HNE inhibition. In the following assay, astragaloside IV (**7**), a tetracyclic triterpene, was used as the negative control.

**Figure 4:**
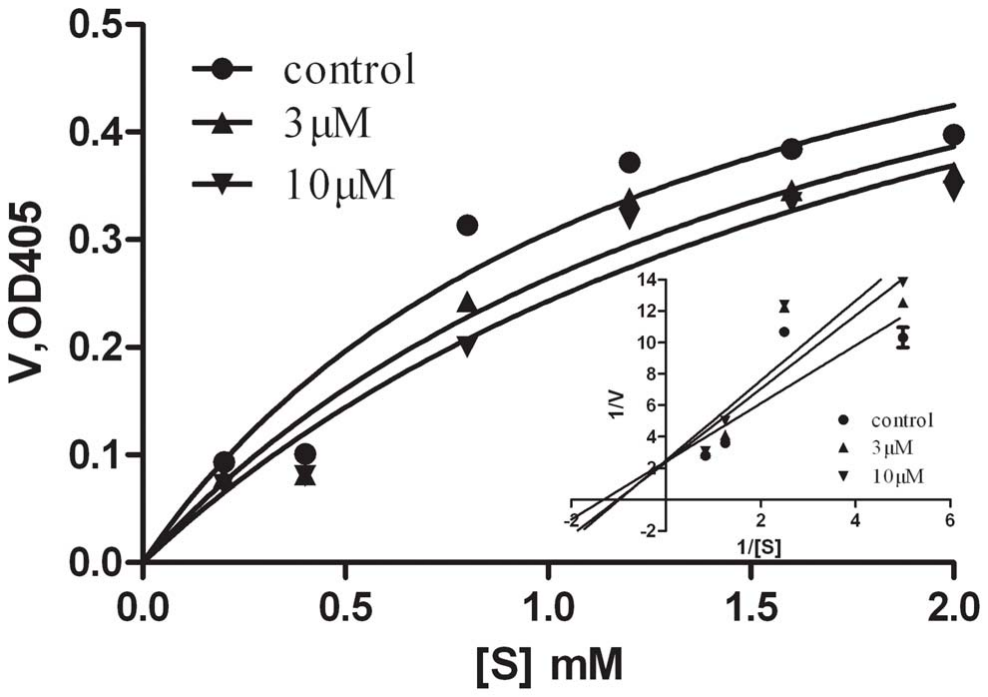
Michaelis-Menten plot and double reciprocal Lineweaver-Burk plot of ursolic acid inhibition of HNE activity. Reactions were carried out at 37°C with various HNE substrate (MeO-Suc-Ala-Ala-Pro-Val-pNA) concentrations in combination with 0, 3, and 10 µM ursolic acid.

The inhibitory kinetics of ursolic acid was determined in the presence of six HNE substrate concentrations (**Fig. 4**). Analysis of the Michaelis–Menten plot showed that K_app_ increased with increasing ursolic acid concentration from 0 µM to 10 µM (P<0.05). The trend of V_max_′ was basically considered to maintain invariable as a function of ursolic acid concentration (P>0.05). These results suggest that ursolic acid competitively inhibits HNE with respect to substrate concentration.

### Activity of Triterpenes in an Acute Smoke Model

**Figure 5:**
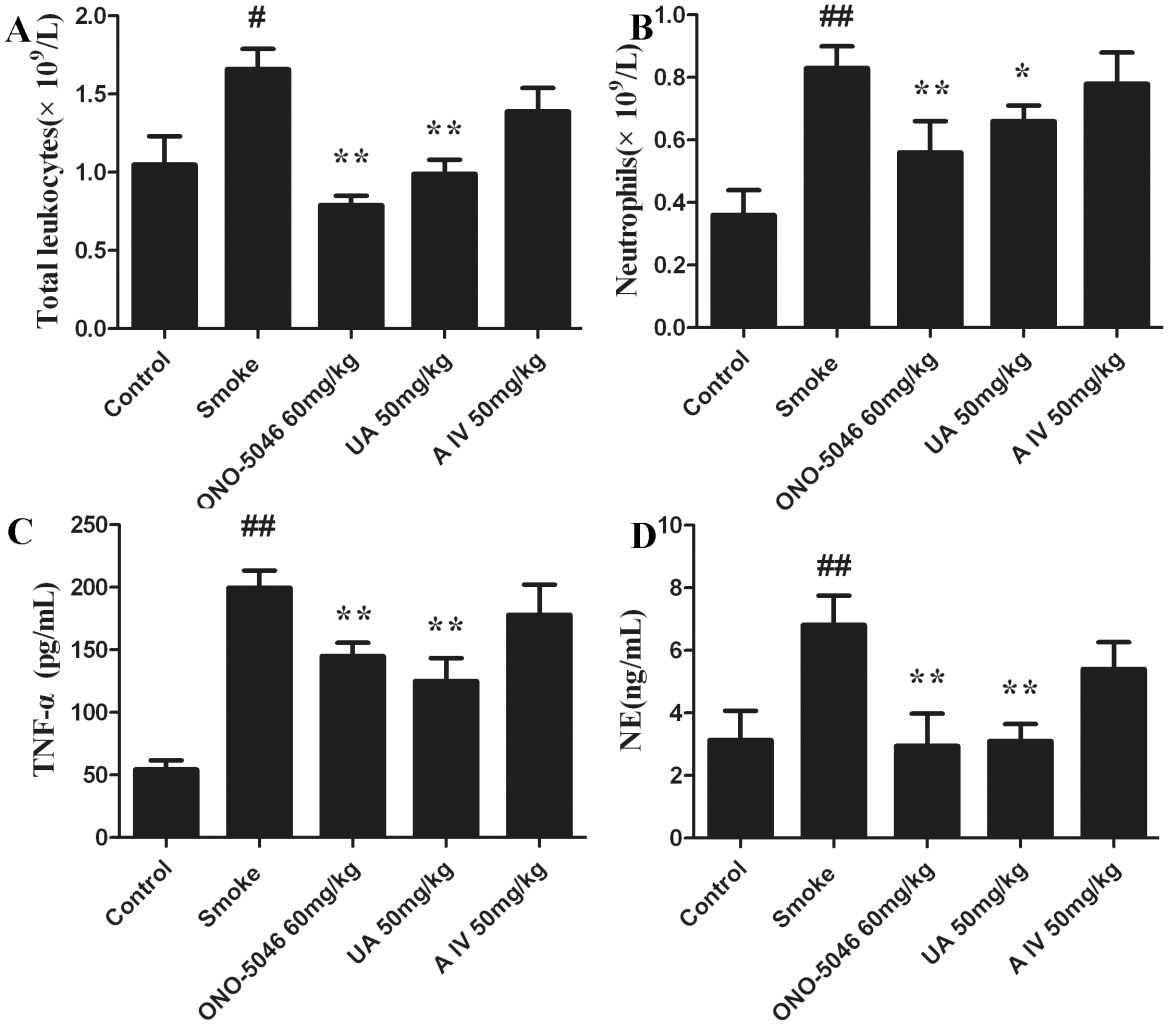
Effects of ONO-5046, ursolic acid (1), and astragaloside IV (7) on tobacco smoke–induced airway inflammation in mice. (A) Total leukocyte change in BALF (B) BALF neutrophils (C) TNF-α levels (D) NE levels. Data are expressed as mean ± standard error (n = 8). ^#^, P<0.05 vs. the control group. ^##^, P<0.01 vs. the control group. *, P<0.05 vs. the smoke group. **, P<0.01 vs. the smoke group. UA: ursolic acid, A IV: astragaloside IV.

**Figure 6:**
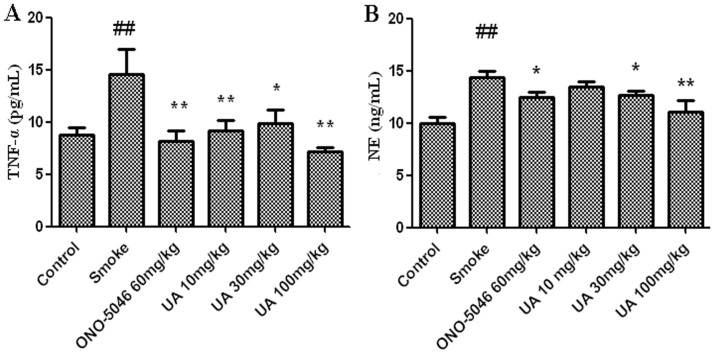
Activity of ursolic acid (compound 1) on acute smoke mouse model. Dose vs. response data for UA measuring BALF TNF-α (A) and HNE (B). Values are expressed as mean ± standard error (n = 8). ##, P<0.01 vs. the control group. *, P<0.05 vs. the smoke group. **, P<0.01 vs. the smoke group. UA: ursolic acid.

In the acute smoke-induced airway inflammation model, the mice exposed to cigarette smoke for 5 d had significantly increased number of total leukocytes and neutrophils in the airway wall (**Figs. 5A and 5B**), but not the lymphocytes (**Fig. S1 in File S1**). In the BALF complement, the TNF-α from activated macrophages and the NE released from the activated neutrophils were augmented after exposure to smoke (**Figs. 5C and 5D**). In the initial animal trial, intraperitoneal injection of 50 mg/kg ursolic acid (**1**) significantly reduced the number of total leukocytes and neutrophils. At the same dose, it also inhibited the increase in BALF NE by 54% and the increase in TNF-α by 38%. We further assayed the effects of ursolic acid (**1**) on airway inflammation in mice at 10, 30, and 100 mg/kg. The results showed that ursolic acid reduces the levels of BALF NE and TNF-α in a dose-dependent manner, with significant inhibition at 30 mg/kg (P<0.05) (**Fig. 6B**). TNF-α was significantly inhibited at ≥10 mg/kg (**Fig. 6A**). Under both treatments, ONO-5046 prevented the smoke-induced increase in BALF inflammatory cells and proinflammatory cytokine levels (**Figs. 5** **and** **6**). However, astragaloside IV (**7**) did not inhibit airway inflammation (**Fig. 5**). Collectively, our results indicate that ursolic acid inhibits smoke-induced NE activity in mouse airways.

### NMR-detected Interaction of the Six Pentacyclic Triterpenes with HNE

NMR evaluation was based on the comparison of the selective (*R^se^*) and nonselective (*R^ns^*) spin-lattice relaxation rates of the ligand with and without the enzyme. The change in the formation of ligand–enzyme adducts affected *R^ns^* and *R^se^* at different extents, and the slower rotational tumbling primarily affected *R^se^* [40], [41]. Upon binding to a macromolecule, the increase in direct relaxation of protons of ligand was averaged mostly by negative cross-relaxation because of slow molecular tumbling, which indicates that *R^ns^* is affected to a lesser extent and that the ***R^ns^*** **/** ***R^se^*** ratio decreased during the binding process. The change in the ***R^ns^*** **/** ***R^se^*** ratio from the ligand with and without the enzyme occurs only when the ligand binds to the enzyme in a fast reversible exchange manner [42]–[44]. Therefore, the decrease in the ***R^ns^*** **/** ***R^se^*** ratio indicates the reversible interaction between the ligand and the enzyme.

The *R^ns^* and *R^se^* of the protons in the pentacyclic triterpenes were measured with and without HNE. After a detailed detection of the ^1^H NMR spectra of compounds **1** to **6**, the high-resolution peaks were chosen for subsequent investigation. Table 2 summarizes the chemical shifts, *R^ns^* and *R^se^*, of the selected protons from compounds **1** to **6** with and without HNE. All chemical shifts were almost unaffected with and without HNE, whereas almost all observed proton relaxation rates were changed with HNE (**Table 2**). Most of the detected protons also increased in *R^se^* with HNE because of the decrease in the *R^ns^*/*R^se^* ratio. The *R^ns^/R^se^* values among the observed protons decreased at different extents. This result can be attributed to the different positions of the investigated protons in the HNE active domain. Therefore, the observed protons interacted differently with the dipolarly connected protons of HNE, thereby changing the *R^ns^/R^se^* value, especially after adding HNE. Comparison of the decrease in the ***R^ns^*** **/** ***R^se^*** of the ligand with and without HNE revealed that compounds **1** to **6** directly bind to HNE in a reversible manner [41].

**Table 2:** ^1^H NMR parameters of six pentacyclic triterpenes (1 mM) at 400 MHz in D_2_O buffered at pH 7.0, T = 298 K with and without HNE (5 µM).

Protons name	Free ligand				Free ligand+HNE			
	*δ* (ppm)	*R^ns^*(s^−1^)	*R^se^*(s^−1^)	*R^ns^/R^se^*	*δ* (ppm)	*R^ns^*(s^−1^)	*R^se^*(s^−1^)	*R^ns^/R^se^*
***Ursolic acid (1)***								
**H_18_**	2.11	2.12	2.31	**0.92**	2.10	2.29	2.83	**0.81**
**H_3_**	2.98	2.07	2.24	**0.93**	2.98	2.40	3.29	**0.73**
**H_12_**	5.00	1.93	2.19	**0.88**	5.00	1.90	3.07	**0.62**
***Oleanolic acid (2)***								
**H_18_**	2.76	2.16	2.52	**0.86**	2.76	2.15	2.63	**0.82**
**H_3_**	2.98	2.04	2.18	**0.86**	2.98	2.01	2.21	**0.91**
**H_12_**	5.03	1.79	2.09	**0.86**	5.03	1.76	2.15	**0.82**
***Glycyrrhetinic acid (3)***								
**H_5_**	0.67	3.23	3.52	**0.92**	0.67	3.19	3.82	**0.84**
**H_18_**	1.98	5.04	2.20	**2.29**	1.98	5.02	6.18	**0.81**
**H_3_**	3.01	2.17	2.25	**0.96**	3.01	2.24	3.04	**0.74**
**H_12_**	5.53	1.31	1.39	**0.94**	5.53	1.36	1.61	**0.85**
***Glycyrrhizin (4)***								
**H_9_**	2.30	2.28	3.57	**0.64**	2.29	2.35	3.67	**0.64**
**glc-H_1’_**	4.29	1.45	2.69	**0.54**	4.28	1.44	4.59	**0.31**
**glc-H_1”_**	4.58	1.32	2.69	**0.49**	4.59	1.27	2.73	**0.47**
**H_12_**	5.41	1.29	1.48	**0.87**	5.53	1.19	1.61	**0.74**
***Betulinic acid (5)***								
**H_24_**	0.61	2.57	2.52	**1.02**	0.62	2.56	2.69	**0.95**
**H_13_**	2.08	3.83	2.53	**1.51**	2.08	3.95	4.06	**0.97**
**H_19_**	3.00	2.07	2.53	**0.82**	3.02	2.07	2.62	**0.79**
**H_29b_**	4.48	2.41	2.48	**0.97**	4.47	2.39	2.47	**0.96**
**H_29a_**	4.60	2.93	3.06	**0.96**	4.59	2.87	3.03	**0.95**
***Lupeol (6)***								
**H_24_**	0.66	2.42	2.35	**1.03**	0.66	2.46	2.39	**1.03**
**H_13_**	1.87	3.68	2.20	**1.67**	1.87	3.60	2.83	**1.27**
**H_19_**	2.98	2.26	2.20	**1.03**	2.98	2.33	2.33	**1.00**
**H_29b_**	4.55	1.98	1.60	**1.24**	4.55	2.05	1.68	**1.22**
**H_29a_**	4.69	2.36	1.91	**1.23**	4.69	2.45	2.00	**1.23**

## Discussion

Through HNE activity inhibitory kinetics with *in vitro* NMR methods, we found that the pentacyclic triterpenes, but not the tetracylic triterpenes, significantly inhibit HNE in a reversible and competitive manner, thereby preventing some side effects caused by the covalent binding of ONO-5046 to HNE, including drug accumulation, hypersensitivity, and increased risk of toxicity [45]. Although both ursolic acid and astrogaloside IV reportedly inhibit NF-κB, only ursolic acid inhibited inflammation *in vivo*. This result can be attributed to the addition of HNE inhibitory activity.

CDDO-Me, which is currently being tested in Phase II clinical trials, prevents and treats lung cancer by inducing the apoptosis of cancer cells in mice [46]. CDDO-Me also reportedly suppresses the inflammatory response and oxidative stress by affecting the nuclear factor kappa B (NF-κB) and the nuclear factor erythroid 2-related factor 2 pathways [37], [47]. We found that CDDO-Me significantly inhibits HNE activity *in vitro*, which could reveal another of its important mechanism of action against lung inflammation.

HNE is a glycoprotein with a single 218–amino acid peptide chain that forms two domains of beta-barrels stabilized by four disulfide bridges (Cys: 42–58, 136–201, 168–182, and 191–220) [48]. Its potent catalytic activity is facilitated by a His57–Asp102–Ser195 catalytic triad that is highly conserved in serine proteases. HNE mainly includes the primary catalytic S_1_ pocket and the extended substrate-binding subsites S_2_ to S_5_, which tend to be hydrophobic [48].

**Figure 7:**
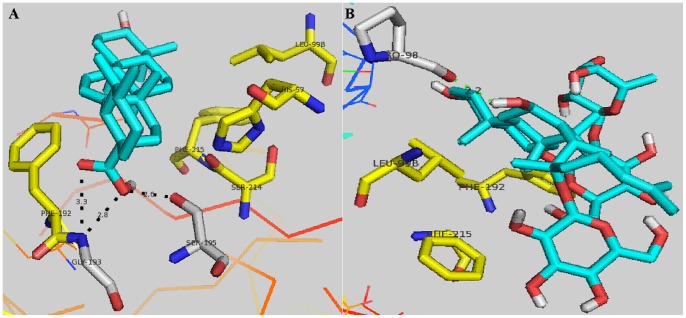
Interaction modes of ursolic acid (A) and astragaloside IV (B) with HNE. For each ligand (blue), a putative binding mode from molecular docking calculations was prepared using PyMol. The important residues for ligand–NE hydrophobic interactions are represented by yellow rod-like structures and hydrogen bonding interactions are represented by gray rod-like structures. Hydrogen bonds are represented by black dotted lines.

The comparison of the docking and bioassay results of the pentacyclic triterpenes **1** to **6** and the tetracyclic triterpenes **7** to **12** showed that the molecular scaffold, the 28-COOH, and a double bond in the appropriate location in their skeleton increased their inhibitory activity. The placement of the ligand into the HNE catalytic pocket explains the need for its hydrophobicity. The six pentacyclic triterpenes selected interacted with the extended substrate-binding domain of HNE at subsites S_3_, covered S_2_, S_4_, and S_5_, and pointed to the oxyanion hole (Gly193 and Ser195) in the S_1_ pocket, whereas the six tetracyclic triterpenes occupied subsite S_2_, subsite S_4_, or none of the subsites. This result suggests that the skeleton shape and the molecular size of the ligand are important in HNE inhibitory activity. Important hydrogen bonds that anchor the pentacyclic triterpenes into the HNE binding site were also identified. Figure 7A shows that the C-28-COOH of ursolic acid (**1**) pointed toward the backbone NHs of Ser195 and Gly193 (the oxyanion hole), and that the distances were suitable for the optimal hydrogen bonding of Ser195 and Gly193 [49]. By contrast, astragaloside IV (**7**) had no hydrogen bonds with the appropriate key residues (**Fig. 7B**). The replacement of the carboxyl group at position 28 with a methyl group, as in lupeol (**6**), reduced the inhibitory potency of its methylation analogue betulinic acid (**5**). This finding indicates that the 28-COOH in the pentacyclic ring system of the triterpenes contribute to HNE binding. The difference in HNE inhibitory activity between oleanolic acid (**2**) and glycyrrhetinic acid (**3**) also suggests the importance of the 28-COOH. The pentacyclic triterpenes, including ursolic acid (**Fig. 7A**), induced hydrophobic interactions with the peptide backbone of Ser214–Val216 of HNE, which is typical of binding to serine proteases. Subsite S_3_ possesses Phe192, which behaves as a receptor site for double bond fragments of hydrophobic pentacyclic triterpene inhibitors [50]. By contrast, the six tetracyclic triterpenes did not occupy subsite S_3_ because of their unsuitable scaffold, such as astragaloside IV (**Fig. 7B**).

The potent inhibitory activity of pentacyclic triterpenes on HNE were supported by the mouse acute smoke-induced lung inflammatory model. The acute smoking model is a relatively sensitive method for investigating lung injury inflammation [29]. In humans and animal models, acute smoke exposure causes tissue damage, as suggested by the elevated levels of lipid peroxidation products and the degradation products of extracellular matrix proteins [51]–[54]. Acute smoke exposure stimulates the recruitment of neutrophils and the release of several inflammatory mediators (e.g., NE, TNF-α, IL-1β, and IL-8) in animal lung tissue and BALF [7], [29]. Therefore, the acute smoke model provides specific information regarding the pathophysiologic mechanisms of lung disorders.

Recent studies have demonstrated that HNE stimulates diverse proinflammatory signaling pathways through multiple mechanisms. Therefore, HNE is an important regulator of inflammatory processes and is an interesting target for new therapeutic approaches against inflammatory disorders, particularly those involving the lungs [7], [55]. The inflammatory response in lung disorders is characterized by high levels of TNF-α, which attracts neutrophils to the lung, thereby causing the excessive release of HNE [56]. In the mouse model of acute smoke-induced inflammation, treatment with ursolic acid significantly reduced the neutrophils and total leukocytes in BALF, which indicates that ursolic acid attenuates the permeability of pulmonary vessels [8]. The reduction in HNE and TNF-α is consistent with previous studies, which show that HNE inhibitors suppress TNF-α secretion from activated macrophages [57], [58]. Moreover, the mice pretreated with endogenous HNE inhibitor α1-AT were unable to secrete TNF-α in response to d-galactosamine with lipopolysaccharide (LPS) and were therefore fully protected against d-galactosamine with LPS-induced hepatitis [59]. Pentacyclic and tetracyclic triterpenes, such as ursolic acid **(1)** and astragaloside IV **(7)**, reportedly have anti-inflammatory and antioxidant functions through multiple mechanisms, such as by inhibiting NF-κB, iNOS, COX-2, and other proinflammatory mediators [60]–[62]. Proinflammatory cytokines such as IL-1β and TNF-α are induced by acute smoke exposure through NF-κB–dependent mechanisms [60]. Ursolic acid significantly suppressed the levels of BALF NE and TNF-α *in vivo*. However, we did not observe the inhibitory effects of astragaloside IV on NE and TNF-α. These results imply that the anti-inflammatory effects of the pentacyclic triterpenes are closely related to direct inhibition of HNE activity. This inhibition reduced the TNF-α level, which prevented neutrophil activation and their subsequent chemotaxis to inflammatory sites with the excessive HNE release.

In conclusion, pentacyclic triterpenes, but not tetracyclic triterpenes, protect against inflammation-induced lung injury by reversibly and competitively inhibiting HNE activity. Furthermore, we deduced that the scaffold, the carboxyl group at position 28, and a double bond at the appropriate location in the pentacyclic ring system of the triterpenes contributed to HNE binding. To the best of our knowledge, this study is the first to demonstrate the structure–activity relationship between pentacyclic and tetracyclic triterpenes, thereby providing insights into the molecular mechanism underlying their pharmacologic effects on lung inflammatory actions. Therefore, compared with synthetic HNE inhibitors, the less toxic pentacyclic triterpenes, which are present in many fruits, have better therapeutic value in preventing HNE-induced inflammation.

## Supporting Information

File S1 **Combined file of supporting figures and tables.**
(DOC)
