# Supplementary material for: Inhibition of Human Neutrophil Elastase by Pentacyclic Triterpenes
Source: PLoS One. 2013 Dec 20;8(12):e82794. doi: 10.1371/journal.pone.0082794 (PMC3869726; doi:10.1371/journal.pone.0082794)
Supplement: File S1 — Combined file of supporting figures and tables. (DOC) [file pone.0082794.s001.doc]

**Supporting information**

**Table S1 The** compound numbers of 11 herbs

| **No.** | **Herb name** | **Numbers** |
| --- | --- | --- |
| 1 | *Hedyotis diffusa* | 8 |
| 2 | *Portulaca grandiflora* | 4 |
| 3 | *Xanthium sibiricum* | 20 |
| 4 | *Ligusticum chuanxiong* | 25 |
| 5 | *Salvia miltiorrhiza* | 19 |
| 6 | *Angelica sinensis* | 70 |
| 7 | *Rehmannia glutinosa* | 66 |
| 8 | *Cordyceps sinensis* | 12 |
| 9 | *Schisandra chinensis* | 34 |
| 10 | *Astragalus membranaceus*(Fisch)Bge | 18 |
| 11 | *Glycyrrhiza uralensis* | 47 |

Table S2 57 compounds selected by molecular docking

| **No.** | **CAS** | **Compound Name** |
| --- | --- | --- |
| 1 | 83-48-7 | Stigmasterol |
| 2 | 57-87-4 | Ergosterol |
| 3 | 508-02-1 | oleanolic acid |
| 4 | 77-52-1 | Ursolic acid |
| 5 | 111150-27-7 | Uralenolide |
| 6 | 98063-18-4 | 24-Hydroxyglabrolide |
| 7 | 471-53-4 | Glycyrrhetinic Acid |
| 8 | 1405-86-3 | Glycyrrhizic Acid |
| 9 | 59-30-3 | Folic acid |
| 10 | 58-05-9 | Folinic acid |
| 11 | 26791-73-1 | Xanthatin |
| 12 | 55555-39-0 | Xanthinosin |
| 13 | 1124-11-4 | chuanxiongzine |
| 14 | 68-19-9 | Vitamin B12 |
| 15 | 86541-79-9 | Astragenol |
| 16 | 474-58-8 | Daucosterol |
| 17 | 83-46-5 | β-sitosterol |
| 18 | 568-72-9 | Tanshinone Ⅱa |
| 19 | 22550-15-8 | Isocryptotanshinone |
| 20 | 33465-16-6 | Huratoxin |
| 21 | 39089-30-0 | Karacoline |
| 22 | 156281-31-1 | Kanzonol L |
| 23 | 134958-55-7 | Gancaonin T |
| 24 | 152511-46-1 | Kanzonol H |
| 25 | 152546-94-6 | Kanzonol I |
| 26 | 134958-52-4 | Gancaonin Q |
| 27 | 83207-60-7 | Astramembrannin II |
| 28 | 134958-56-8 | Gancaonin U |
| 29 | 69-65-8 | D-mannitol |
| 30 | 26166-37-0 | denudatine |
| 31 | 89354-45-0 | (Z)-6,8'7,3'-Diligustilide |
| 32 | 93236-64-7 | Wallichilide |
| 33 | 106533-38-4 | 3,8-Dihydro-6,6’,7,3’a-diligustilide |
| 34 | 27303-26-0 | Libanorin |
| 35 | 88182-33-6 | Diligustilide |
| 36 | 10191-41-0 | Vitamin E |
| 37 | 39089-30-0 | Carmicheline |
| 38 | 152511-46-1 | Kanzonol H |
| 39 | 142182-61-4 | Senkyunone |
| 40 | 152511-47-2 | Kanzonol J |
| 41 | 6921-64-8 | 2’-Hydroxy-4’-methylacetophenone |
| 42 | 35825-57-1 | Cryptotanshinone |
| 43 | 115841-09-3 | Salvianolic acid C |
| 44 | 568-73-0 | Tanshinone I |
| 45 | 17397-93-2 | Tanshinone IIB |
| 46 | 43043-07-8 | Angeladin |
| 47 | 23027-48-7 | Decursidin |
| 48 | 59015-74-6 | Eicosanoyl-Stigmast-5-en-3-ol |
| 49 | 5928-25-6 | Desursin |
| 50 | 96608-82-1 | Edulisin I |
| 51 | 132998-82-4 | Xanthoangelol C |
| 52 | 21174-75-4 | Archangelin |
| 53 | 59-43-8 | Vitamin B1 |
| 54 | 98063-17-3 | 3,24-Dihydroxy-11,13(18)-oleanadien  -30-oic acid |
| 55 | 156281-31-1 | Kanzonol L |
| 56 | 98063-18-4 | 24-Hydroxyglabrolide |
| 57 | 152546-94-6 | Kanzonol I |

**Table S3 Inhibition of HNE activity of 6 tetracyclic triterpenoids at 100μ**M

| **Compound** | **Inhibition (%)** |
| --- | --- |
| **7** | **3.27** |
| **8** | **4.01** |
| **9** | **5.77** |
| **10** | **2.33** |
| **11** | **4.28** |
| **12** | **0.80** |
